# Supplementary material for: Genetic differences in host infectivity affect disease spread and survival in epidemics
Source: Sci Rep. 2019 Mar 20;9:4924. doi: 10.1038/s41598-019-40567-w (PMC6426847; doi:10.1038/s41598-019-40567-w)
Supplement: Supplementary file 1 — Supplementary Infoormation [file 41598_2019_40567_MOESM1_ESM.docx]

**Supplementary Information**

**Genetic differences in host infectivity affect disease spread and survival in epidemics**

Osvaldo Anacleto^1^**,** Santiago Cabaleiro^2^, Beatriz Villanueva^3^, María Saura^3^, Ross D. Houston^1^, John A. Woolliams^1^, Andrea B. Doeschl-Wilson^1^

1The Roslin Institute and Royal (Dick) School of Veterinary Studies, University of Edinburgh, UK

^2^Centro Tecnológico del Cluster de la Acuicultura (CETGA), Spain

^3^Departamento de Mejora Genética Animal, INIA*,* Spain

Supplementary Figure 1. Tank differences in onset of visual signs and survival post disease. Evolution of disease caused by *Philasterides dicentrarchi* (a-b) and survival post disease (c-d) in all families of recipient fish in trials 1 (T1) and 2 (T2) of the transmission experiment. The curves were obtained through tank-based Kaplan-Meier plots for time to signs (a-b) and time from signs to death (c-d). P-values were calculated using the two tailed log-rank test for detecting family differences in Kaplan-Meier estimates.

**Supplementary Figure 2. Survival of shedder fish families.** Kaplan-Meier curves for time to death of shedder fish for trials 1 (a) and 2 (b), by family. Most infective shedder families were C and F for trials 1 and 2, respectively. Least infective shedder families were B and G for trials 1 and 2, respectively. P-values were calculated using the two tailed log-rank test for detecting family differences in Kaplan-Meier estimates

**Supplementary Table 1.** Number of fish from each of the trial 1 recipient families shown in Kaplan-Meier plots in Figures 4a and 4c.

| **family** | **time to disease signs** | | |  | **time from signs to death** | | |
| --- | --- | --- | --- | --- | --- | --- | --- |
|  | **event** | **censored** | **total** |  | **event** | **censored** | **total** |
| 7 | 12 | 27 | 39 |  | 12 | 0 | 12 |
| 8 | 30 | 3 | 33 |  | 26 | 4 | 30 |
| 10 | 28 | 8 | 36 |  | 26 | 2 | 28 |
| 11 | 26 | 9 | 35 |  | 23 | 3 | 26 |
| 12 | 26 | 10 | 36 |  | 24 | 2 | 26 |
| 13 | 28 | 6 | 34 |  | 27 | 1 | 28 |
| 14 | 21 | 4 | 25 |  | 18 | 3 | 21 |
| 15 | 25 | 9 | 34 |  | 21 | 4 | 25 |
| 16 | 23 | 11 | 34 |  | 23 | 0 | 23 |
| 18 | 25 | 2 | 27 |  | 24 | 1 | 25 |
| 19 | 21 | 15 | 36 |  | 18 | 3 | 21 |
| 20 | 13 | 14 | 27 |  | 12 | 1 | 13 |
| 22 | 26 | 7 | 33 |  | 26 | 0 | 26 |
| 28 | 26 | 3 | 29 |  | 26 | 0 | 26 |
| 30 | 13 | 20 | 33 |  | 11 | 2 | 13 |
| 32 | 25 | 7 | 32 |  | 21 | 4 | 25 |
| 33 | 27 | 8 | 35 |  | 24 | 3 | 27 |
| 39 | 25 | 9 | 34 |  | 20 | 5 | 25 |
| **Total** | **420** | **172** | **592** |  | **382** | **38** | **420** |

**Supplementary Table 2.** Number of fish from each of the trial 2 recipient families shown in Kaplan-Meier plots in Figures 4b and 4d.

| **family** | **time to disease signs** | | |  | **time from signs to death** | | |
| --- | --- | --- | --- | --- | --- | --- | --- |
|  | **event** | **censored** | **total** |  | **event** | **censored** | **total** |
| 9 | 16 | 14 | 30 |  | 16 | 0 | 16 |
| 36 | 21 | 11 | 32 |  | 21 | 0 | 21 |
| 38 | 14 | 18 | 32 |  | 14 | 0 | 14 |
| 42 | 17 | 9 | 26 |  | 17 | 0 | 17 |
| 43 | 19 | 18 | 37 |  | 19 | 0 | 19 |
| 45 | 18 | 13 | 31 |  | 17 | 1 | 18 |
| 47 | 29 | 5 | 34 |  | 28 | 1 | 29 |
| 48 | 25 | 9 | 34 |  | 25 | 0 | 25 |
| 52 | 16 | 20 | 36 |  | 16 | 0 | 16 |
| 53 | 19 | 10 | 29 |  | 18 | 1 | 19 |
| 54 | 16 | 21 | 37 |  | 15 | 1 | 16 |
| 55 | 19 | 11 | 30 |  | 19 | 0 | 19 |
| 56 | 23 | 13 | 36 |  | 23 | 0 | 23 |
| 58 | 17 | 18 | 35 |  | 16 | 1 | 17 |
| 59 | 19 | 10 | 29 |  | 19 | 0 | 19 |
| 60 | 19 | 15 | 34 |  | 19 | 0 | 19 |
| 63 | 9 | 23 | 32 |  | 9 | 0 | 9 |
| 70 | 26 | 8 | 34 |  | 26 | 0 | 26 |
| **Total** | **342** | **246** | **588** |  | **337** | **5** | **342** |

**Supplementary Table 3.** Number of recipient fish exposed to each of the eight shedder families shown in Kaplan-Meier plots in Figure 5.

| **shedder family** | **time to disease signs** | | |  | **time from signs to death** | | |
| --- | --- | --- | --- | --- | --- | --- | --- |
|  | **event** | **censored** | **total** |  | **event** | **censored** | **total** |
| 23 | 118 | 35 | 153 |  | 102 | 17 | 119 |
| 24 | 73 | 71 | 144 |  | 63 | 10 | 73 |
| 25 | 127 | 18 | 145 |  | 123 | 4 | 127 |
| 27 | 101 | 48 | 149 |  | 94 | 7 | 101 |
| 31 | 93 | 51 | 144 |  | 92 | 1 | 93 |
| 44 | 111 | 45 | 156 |  | 107 | 3 | 110 |
| 46 | 57 | 85 | 142 |  | 56 | 1 | 57 |
| 51 | 82 | 65 | 147 |  | 82 | 0 | 82 |
| **Total** | **762** | **418** | **1180** |  | **719** | **43** | **762** |
